# Fast Fitting of the Dynamic Memdiode Model to the Conduction Characteristics of RRAM Devices Using Convolutional Neural Networks

**DOI:** 10.3390/mi13112002

**Published:** 2022-11-17

**Authors:** Fernando Leonel Aguirre, Eszter Piros, Nico Kaiser, Tobias Vogel, Stephan Petzold, Jonas Gehrunger, Timo Oster, Christian Hochberger, Jordi Suñé, Lambert Alff, Enrique Miranda

**Affiliations:** 1 Departament d’Enginyeria Electrònica, Universitat Autònoma de Barcelona, 08193 Cerdanyola del Vallès, Spain; 2 Advanced Thin Film Technology Division, Institute of Materials Science, Technische Universität Darmstadt, 64289 Darmstadt, Germany; 3 Computer Systems Group, Department of Electrical and Information Engineering, Technische Universität Darmstadt, 64289 Darmstadt, Germany; 4 Integrated Electronic Systems, Department of Electrical and Information Engineering, Technische Universität Darmstadt, 64289 Darmstadt, Germany

**Keywords:** RRAM, neural networks, curve fitting, dynamic memdiode, memristor

## Abstract

In this paper, the use of Artificial Neural Networks (ANNs) in the form of Convolutional Neural Networks (AlexNET) for the fast and energy-efficient fitting of the Dynamic Memdiode Model (DMM) to the conduction characteristics of bipolar-type resistive switching (RS) devices is investigated. Despite an initial computationally intensive training phase the ANNs allow obtaining a mapping between the experimental Current-Voltage (*I-V*) curve and the corresponding DMM parameters without incurring a costly iterative process as typically considered in error minimization-based optimization algorithms. In order to demonstrate the fitting capabilities of the proposed approach, a complete set of *I-V*s obtained from Y_2_O_3_-based RRAM devices, fabricated with different oxidation conditions and measured with different current compliances, is considered. In this way, in addition to the intrinsic RS variability, extrinsic variation is achieved by means of external factors (oxygen content and damage control during the set process). We show that the reported method provides a significant reduction of the fitting time (one order of magnitude), especially in the case of large data sets. This issue is crucial when the extraction of the model parameters and their statistical characterization are required.

## 1. Introduction

Since the first practical description of a memristive device by HP in 2008 [1], a number of compact models for the current-voltage (*I-V*) characteristic of resistive switching (RS) devices have been proposed [2]. However, selecting an appropriate generic model for an electron device is far from being a simple and direct task. The model should not only be able to cover the basic features experimentally observed, but also the distinctive details of the device under study. The accurate representation of the electron transport mechanism in the investigated device encourages the design and simulation of more complex circuits and/or systems and allows the identification and organization of the elementary pieces that lead to the variety of observed behaviors. For circuit simulation-oriented models, this capacity of adaptation must be achieved by means of a reduced number of parameters and by well-behaved and selected equations. This is the signature of a compact behavioral approach: the emphasis is on the phenomenon representation rather than the physical details behind it. This does not mean that physical aspects are completely abandoned, but they must be conjugated with suitable ad hoc concepts and appropriate mathematical tools. Among this kind of behavioral approaches, the Dynamic Memdiode Model (DMM) proposed by our group in Ref. [2] has been proven capable not only of simulating isolated RRAM devices, but also simulating large RRAM-based Artificial Neural Networks (ANN) with parasitic elements used for pattern recognition tasks [3,4].

Nonetheless, the fitting of parametrized functional forms, such as those involved in the DMM, to sets of experimental data (curve fitting) is a well-known problem in data analysis. The optimal parameter values are conventionally found by minimizing an error measure, often taken to be the sum of the squares of the errors between the observed data values and those predicted by the function. If the functional form is linearly dependent on the parameters (e.g., a polynomial), then the minimization problem is linear and can be easily solved. However, in many cases, it is necessary to consider functional forms which nonlinearly depend on the unknown parameters. The error minimization procedure in such cases generally involves an iterative algorithm starting from an initial guess. Depending on the number of parameters, such iterative methods can be computationally intensive and hence, slow and for complex problems, the selection of a suitable initial guess can require human intervention to ensure convergence to the correct solution. This is the case of the Levenberg–Marquardt (LM) [5,6,7] and Genetic (GA) algorithms [8,9,10,11] which have been proposed to automate the model parameter calibration (extraction). While the LM method tends to be stuck in a local optimum when launched without a proper initial guess, the GA is a global optimizer for combinatorial optimization problems, but it is not recommended for tuning continuous parameters. In addition, the Pattern Search (PS) algorithm (one of the so-called direct searching algorithms) [12,13,14] has been considered, as it is a very simple and fast local searching algorithm since it is derivative-free and depends only on simple calculations. Although this method has the capability to step over the hillocks in the parameter space to escape the local optima, due to the independence from derivation in the pattern vector, it loses information to a certain extent in high dimensional problems since it does not utilize any information gained along the optimization trajectory. As a result, for applications involving high volumes of data or for real-time applications, there is considerable interest in techniques which can automate the curve fitting process and operate at high speed [15] without incurring too much energy consumption.

In this paper, we consider the use of convolutional neural networks (CNN) as suitable and fast alternatives to determine the optimal DMM parameter values directly from the raw data [15]. ANNs, such as the CNNs, are widely used in many disciplines such as system identification, control, pattern recognition, gaming, translation, medical diagnosis, finance, etc. They are particularly important for their ability to reproduce and model nonlinear processes. Despite the computational-intensive training phase, which is ideally performed just once, ANN can infer results without the need of an iterative algorithm. Such ANN abilities make this kind of approach much faster than the iterative methods and does not require an initial guess for the solution. Furthermore, for real-time applications, it is possible to implement the network in special purpose hardware, thereby exploiting the intrinsically parallel nature of neural networks and thus, achieving very high processing speeds. To account for the coupled nature of the current and voltage signals in memristive devices, we consider an AlexNET [16] architecture and train it to estimate the DMM parameters from a graphical representation of the *I-V* loops. From the results reported, we show that following this approach, the DMM model accurately fits the experimental data measured under different conditions at a reduced computational cost compared with the standard error minimization method.

## 2. Materials and Methods

### 2.1. Device Fabrication and Electrical Characterization

**Figure 1:**
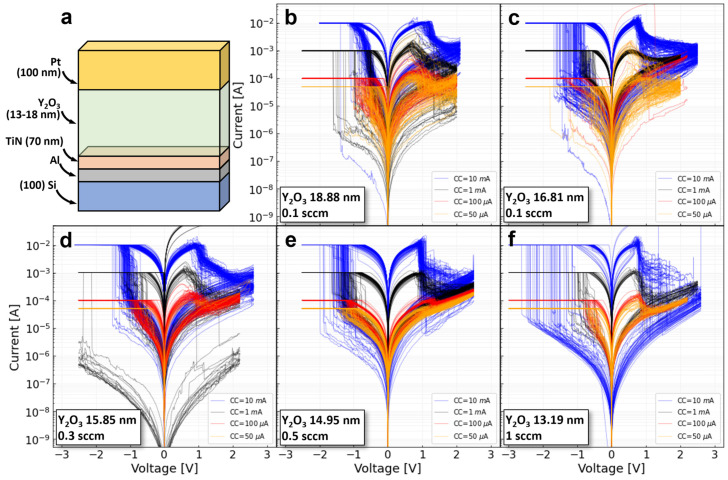
(**a**) Structure of the RRAM devices under test. The insulating layer varies from 13 to 18 nm depending on the oxidation condition. Measured *I-V* loops from current compliances 50 µA, 100 µA, 1 mA, and 10 mA: (**b**) oxygen flow of 0.1 sccm, (**c**) oxygen flow of 0.2 sccm, (**d**) oxygen flow of 0.3 sccm, (**e**) oxygen flow of 0.5 sccm, (**f**) oxygen flow of 1 sccm.

The RRAM devices being tested consist of Si/Al/TiN/Y_2_O_3_-x/Pt stacks as schematically illustrated in Figure 1a. Oxygen-engineering [17] of the functional layer was carried out using a reactive molecular beam epitaxy setup that controls the oxygen stoichiometry of the yttria film by varying the flow of oxygen radicals and the film growth rate. A total of 5 different combinations of oxygen flow and growth rate were considered, including (i) an oxygen flow of 0.1 square cubic centimeter (sccm) with a growth rate of 1 angstrom per second (Å s^−1^), (ii) 0.2 sccm and 1 Å s^−1^, 0.3 sccm and 1 Å s^−1^, 0.5 sccm and 1 Å s^−1^ and 1 sccm and 0.25 Å s^−1^. For a detailed description of the fabrication process and the structural characterization of the samples, please refer to [18]. The electrical characterization was carried out with a Keithley 4200 semiconductor characterization system (SCS) biasing the Pt top electrode and grounding the TiN bottom electrode. The internal current compliance (CC) of the SCS was used to prevent the hard breakdown of the oxide layer during electroforming and SET. In addition, four different compliance levels were considered, and the *I-V* characteristics were measured for over 100 consecutive cycles for each fabrication condition and current compliance level (Figure 1b–f).

### 2.2. Dynamic Memdiode Model (DMM)

**Figure 2:**
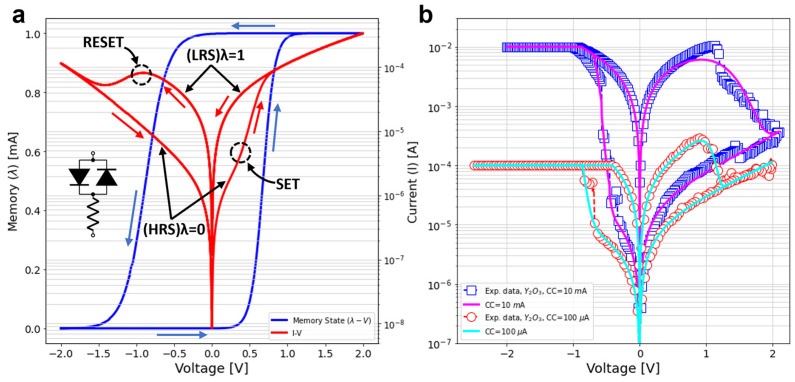
(**a**) Memory state (λ, in blue) as a function of the applied voltage, defined by Equation (2). The inset on the left shows the equivalent circuit model for the current equation (Equation (1) including the series resistance. The diodes are driven by the memory state of the device and one diode is activated at a time. Typical *I-V* characteristic for a memdiode obtained via simulation of the proposed model are superimposed. Current evolution is indicated by the blue arrows. (**b**) Experimental *I-V* loops of Y_2_O_3_ devices fabricated with an oxygen flow of 0.1 sccm and measured with current compliances of 10 mA (blue squares) and 100 μA (red circles), both fitted with the DMM model.

The resistive switching (RS) mechanism is the fundamental physical phenomenon behind ReRAM devices. In the particular cases of CBRAMs (conducting bridge random access memories) and OxRAMs (oxide-based random-access memories), RS relies on the displacement of metal ions/oxygen vacancies within the dielectric film in a Metal-Insulator-Metal (MIM) structure. The displacement is caused by the application of an external electrical stimulus, current or voltage [19,20,21,22]. The migration of ions originates from the alternate completion and destruction of a conductive filament (CF) spanning across the insulating film. For a ruptured CF, the device is in the high resistance state (HRS), often described by an exponential *I-V* relationship, while the completion of the CF leads to the low resistance state (LRS), which often exhibits a linear *I-V* curve [23,24]. In between these two extreme situations, the modulation of the CF transport properties renders intermediate states by voltage-controlled redox reactions. From the modelling viewpoint, the DMM is able to describe the major and minor *I-V* loops and the gradual transitions in bipolar resistive switches for a wide variety of memristive systems. This is accomplished, as shown in the inset of Figure 2a, by considering a nonlinear transport equation based on two identical opposite-biased diodes in series with a resistor. The *I-V* relationship resembles a diode with memory and that is why this device was termed memdiode. Notice that the anti-parallel connected diodes allow the bi-directional current flow through the device; as for both positive and negative polarities, there will always be a forward biased diode. For the sake of completeness, the DMM is succinctly reviewed in the next paragraphs.

Physically, the memdiode is associated with a potential barrier that controls the electron flow in the CF. The conduction properties of this non-linear device change according to the variation of this barrier. Since the area of the CF is uncertain, instead of the potential barrier height, the diode current amplitude is used as the reference variable. Following Chua’s memristive device theory, the memdiode model comprises two equations: one for the electron transport (transport equation, TE) and a second equation for the memory state of the device (memory equation, ME). The transport equation can be derived from the quantum point-contact (QPC) model [25,26,27,28], which uses the finite-bias Landauer approach for the calculation of the current flow in a nanosized filamentary structure [29]. For a wide constriction formed by many conducting channels, neither the number *N* of elemental filamentary structures involved is known, nor can their specific potential barrier parameters (width $\theta_{i}$ and height $\varphi_{i}$) be accessed individually [30]. Therefore, we consider the following heuristic approximation for TE which can be derived from the QPC model:

(1) $$I\left(V_{C}\right)=I_{0}\left(\lambda \right)sinh\left[\alpha \left(\lambda \right)\left(V_{C}-R_{S}\left(\lambda \right)I\right)\right]$$

where *I*_0_(*λ*) = *I_min_*(1 − *λ*) + *I_max_λ* is the diode current amplitude, α a fitting constant, and *R_S_* a series resistance. *I_min_* and *I_max_* are the minimum and maximum values of the current amplitude, respectively. While $R_{S}$ in (1) accounts for the contact resistance, the hyperbolic sine function expresses the barrier resistance. Making such an approximation to avoid the use of the complete Landauer formula is reasonable as Equation (1) has the same functional asymptotes for large $I_{0}$ values $\left(R_{S}I\approx V_{C}\right)$ and low applied voltages $\left(R_{S}I\ll V_{C}\right)$. As *I_0_* increases in Equation (1), the *I-V* curve changes its shape from exponential to linear through a continuum of states as experimentally observed for these kinds of devices. For the sake of completeness, α and $R_{S}$ in (6) receive a similar treatment in the LTSpice script as that given to $I_{0}\left(\lambda \right)$. Both parameters can be swept from a minimum (OFF) to a maximum (ON) if required. If not required, α and $R_{S}$ remain fixed.

*λ* is a control parameter that represents the memory state of the device and runs from 0 (HRS) to 1 (LRS) and vice versa. The ME is conveniently described by the following differential equation [31]:

(2) $$\frac{d\lambda }{dt}=\frac{1-\lambda }{\tau_{S}\left(\lambda ,V\right)}-\frac{\lambda }{\tau_{R}\left(\lambda ,V\right)}$$

where $\tau_{S,R}$ are characteristic times associated with the SET (*V* > 0) and RESET (*V* < 0) transitions, i.e., with the ionic/defect movement within the dielectric film in one or the opposite direction; in the framework of this model, they are represented as:

(3) $$\tau_{S}\left(\lambda ,V\right)=e^{-\eta_{S}\left(V-V_{S}\left(\lambda \right)\right)}$$

(4) $$\tau_{R}\left(\lambda ,V\right)=e^{\eta_{R}\lambda^{\gamma }\left(V-V_{S}\right)}$$

where $\eta_{S,R}$ and $V_{S,R}$ are the transition rates ($\eta_{S}$, $\eta_{R}>0$) and the reference switching voltages ($V_{S}>0$, $V_{R}<0$), respectively. The exponential dependences of (3) and (4) on *V* are a consequence of the ionic/vacancy dynamics associated with the hopping mechanism [32]. The modelling of the snap back (*V_s_*(*λ*) = *V_s_*) and snap forward (γ = 0) features of the *I-V* loops are de-activated for the sake of simplicity [33], mainly due to the fact that the current compliance in the SET partly masks the snap back effect. The combination of Equations (1) and (2) results in an *I-V* loop such as the one that is superimposed to the *λ-V* characteristic illustrated in Figure 2a, which starts in HRS (*λ* = 0) and evolves as indicated by the blue arrows. Another relevant feature of the proposed model is that it can be described by a simple SPICE script as shown in Ref. [3]. Finally, the accuracy of the model is reported in Figure 2b by fitting some of the loops reported in Figure 1. In summary, the proposed DMM not only provides a simple SPICE-compatible implementation for the resistive memory devices, but also a versatile one, as it can accurately fit the major and minor *I-V* loops measured in a wide variety of RRAM devices [33].

### 2.3. Convolutional Neural Networks (CNNs)

**Figure 3:**
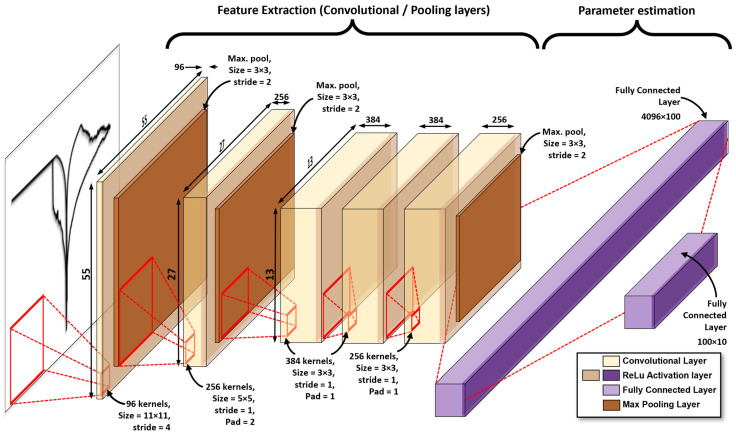
Structure of the convolutional neural network (Alex-NET) used to estimate the DMM fitting parameters.

Besides their use in classification and prediction tasks, neural networks were also considered for parameter estimation in mathematical models. This is the case of the works by Dua [34], Morshed et al., Parikh et al. [35], Gonçalves et al. [36] and Rudi et al. [37], among others, where different kinds of neural networks were explored (Fully Connected Neural Networks—FCNN, Convolutional Neural Networks—CNN, Generative Adversarial Networks—GAN, etc.) for processing time series. Notably, in most of these scenarios, if not all, authors considered user-generated data with a constant sampling rate (invariant number of data points). However, experimental data from *I-V* measurements considering different maximal/minimal amplitudes inevitably implies waveforms with a variable number of datapoints. This is also dictated by the internal electronics of the SCS. Moreover, *I-V* loops contain information from two different time series, that is, current and voltage as a function of time. While it would be possible to consider a CNN for multivariate time series analysis, in our case, we opted for a CNN that estimates the DMM parameters directly from a graphical representation of the *I-V* loop, similar to the classification of audio tracks in terms of their respective spectrogram representation [38]. This has two advantages over the use of the raw measured current and voltage signals: first, we avoid interpolating the data to fit a fixed sized array length, and second, the necessity of the normalization step required for the input signals is eliminated, which, given the exponential nature of the current signal, can be cumbersome from the practical viewpoint (logarithm of negative numbers for raw data). The input to the neural network used in this work consists of images in a constant resolution (227 × 227 pixels) containing the *I-V* curves generated by considering fixed axes for all loops. Based on these images, the CNN responds with a vector of floats normalized in the range (0,1) representing the 10 fitting parameters of the DMM. Because of the big difference between *I_max_* and *I_min_* parameters, the approach described above is recommended. The CNN considered here is a variation of the AlexNET [16] network and whose structure is depicted in Figure 3. The network was trained using the Adam Optimizer Algorithm, while also considering the minimization of the mean squared error as the loss function for over 50 epochs. Dropout layers of 50% were included before the fully connected layers to reduce overfitting.

It is worth mentioning that the choice of the AlexNET over other CNNs with a recognized higher accuracy, (such as the more recent ResNET) responds to the fact that our main target was not to find the best network in terms of accuracy but to provide a fast and energy efficient way to adjust the parameters of a memristor SPICE compact model. For this reason, we searched for a trade-off between the accuracy and power consumption of the neural network training/inference processes. In this connection, it has been shown by Yang et al. [39] that considering energy-aware pruning techniques, ResNET networks incur higher (up to one order of magnitude) energy consumption than their AlexNET counterparts. This is due to the intrinsic, greater depth of the ResNET networks, which results in more feature maps than in an AlexNET CNN that needs to be moved from the storage to the arithmetic unit(s) used for data computation and back to the storage. A breakdown of the energy consumption [39] reveals that most of the energy is used for such data movement and that the amount of data transferred increases with the number of feature maps. This also makes deep ResNETs much slower than the AlexNETs [40]. As such, ResNETs and other advanced CNNs, although capable of achieving higher accuracies, have proven to be not as convenient in terms of energy efficiency.

### 2.4. Database Generation

The database used in this work consists of a collection of 1200 *I-V* loop images generated from pairs of experimentally measured *I*(*t*) and *V*(*t*) signals (with a variable number of points for each loop) plotted as *I*(*t*) vs. *V*(*t*) on a fixed *x*-axis running from −3 V to 3 V, and a fixed *y*-axis running from 1 nA up to 100 mA. The *I-V* images were stored with a fixed resolution of 227 × 227 px. in grayscale. Each image was then accompanied by a 10-element vector containing the normalized DMM parameters (indicated in Section 2.2) that fits the measured data, which acted as the image label in a supervised training procedure. The entire database is partitioned in a 5 to 1 ratio, thereby producing 1000 image-parameter pairs for the training phase and 200 image-parameter pairs for the test phase.

The most challenging part of constructing such a database is obtaining the fitting parameter for each of the 1200 *I-V* loops. Manually fitting this number of *I-V* curves, with a model comprising up to 10 parameters, is impractical and prone to errors. Therefore, we developed an open-source, simulator-in-the-loop (NGSPICE) Python-approach to systematize the data fitting procedure. Files containing the measured data were first parsed and stored in an SQLite database (DB). Then, each *I-V* loop was individually analyzed to extract the so-called “observable parameters”, such as the HRS, LRS, and HRS/LRS ratios, and fitted. The fitting phase can be split into 3 parts:

(i) The *I-V* loop data is divided into 4 segments: HRS region (from the maximal voltage during the RESET *Vmax*→0, ① in Figure 1c), LRS region (from the minimal volage applied during SET, *Vmin*→0, ②), SET region (from 0→*Vmax*, ③), and RESET region (from 0→*Vmin*, ④).
(ii) Each previously mentioned segment is fitted using an approximation of the DMM mathematically derived for the region of interest (and thus neglecting or keeping constant the parameters associated with out-of-scope regions).
(iii) Considering the previous fitting as an initial guess, the optimum parameter values are found by numerical optimization, consisting in an iterative simulation with the SPICE version of the DMM model.
(iv) For performance comparisons, which are described later in the paper, the time required to fit each loop was recorded.

### 2.5. ANN-Based DMM Fitting Procedure

**Figure 4:**
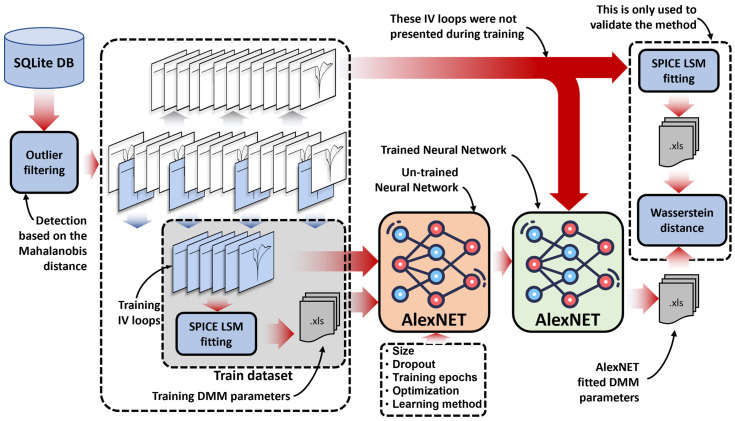
Flowchart of the proposed fitting procedure, including the training of the fitting network. The distribution of predicted values is analyzed in the following sections to discuss the fitting accuracy.

**Figure 5:**
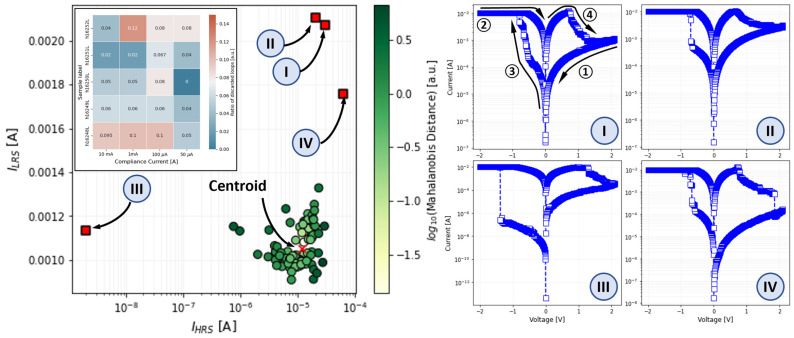
Example of the outlier detection using the Mahalanobis distance. Note that in this example we have considered only 2 variables for the sake of a graphical representation and 4 anomalous cases were found (indicated in red) for which the resulting *p*-value was above 0.1.

The procedure to obtain the best fitting parameters for a given experimental *I-V* loop is described in Figure 4. The starting point is the SQL database of measured *V*(*t*) and *I*(*t*) signals, which are first filtered based on the Mahalanobis distance [41] (with *p* = 0.01). This is carried out to discard anomalous curves (see Figure 5 for the case of samples fabricated with an oxygen flow of 0.1 sccm and measured with a 10 mA current compliance). *V*(*t*) and *I*(*t*) signals are then plotted together as indicated in Section 2.4. At this point, two different scenarios should be distinguished. On one hand, and with the purpose of developing/testing this methodology, the dataset described in Section 2.4 is considered. In this context, the CNN is trained following a supervised approach, and tested based on the previously fitted 1000 and 200 *I-V* loops, respectively. On the contrary, in a real case application scenario, only a fraction of the total *I-V* loops is fitted using the simulator-in-the-loop fitting procedure with the purpose of generating the dataset needed to train the CNN. The DMM parameters for the remaining measurements are then estimated using the trained CNN.

For the first scenario (the one covered by this work), the network performance for fitting the DMM parameters is evaluated based on two metrics: First, the distribution of the fitted DMM parameters using the CNN is compared with the distribution of the corresponding parameters obtained with the simulator-in-the-loop fitting procedure using the Wasserstein distance [41]. Second, the mean squared error (MSE) between the *I-V* loops generated by the predicted DMM parameters and the target *I-V* loops are evaluated. The results obtained following this procedure are reported in the next section.

## 3. Results and Discussion

**Figure 6:**
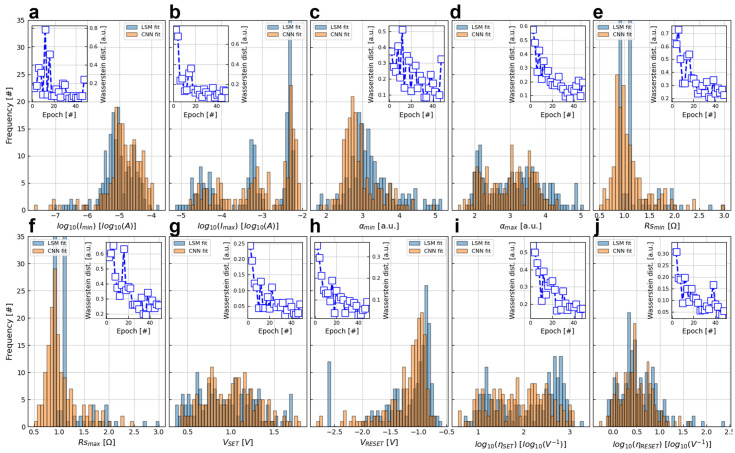
Comparison of the histograms of the estimated DMM parameters using the ANN (labelled as CNN fit) against the DMM parameters extracted by the simulator-in-the-loop approach (labelled as LSM fit). The ANN was trained over 50 epochs. Note the good match between both histograms. The Wasserstein distance in the inset quantifies the fitting improvement along the training.

In this section, the accuracy of the predicted DMM parameter values with the two different metrics described in the previous section is assessed. Let us first consider the superposition of the histograms corresponding to the DMM parameters obtained from the test database (used as labels or target values in the supervised learning approach here considered) with the predicted DMM parameter values obtained by the ANN under study after 50 training epochs (see Figure 6a–j). Note that the predicted values follow very similar distributions when compared to the experimental ones; despite an occasional minor shift in the mean, the standard deviation is fully consistent as is the shape of the distribution. For instance, the 3 peaks observed in the histogram of the target *I_max_* values are nicely reproduced by the *I_max_* values estimated by the CNN. Similarly, the positive and negative skew observed in the histogram of the target α_min_ and *V_RESET_* values are well captured by their CNN fitted counterpart. However, such a nice matching is highly sensitive to the number of training epochs, as it can be seen by the Wasserstein distance vs. number of epochs presented in the inset of each panel in Figure 6. Interestingly, not every parameter has the same sensitivity. In opposition to the gradual reduction observed in the rest of the parameters, the Wasserstein distance for *I_max_* drops abruptly at the very first training epoch and then remains relatively constant.

**Figure 7:**
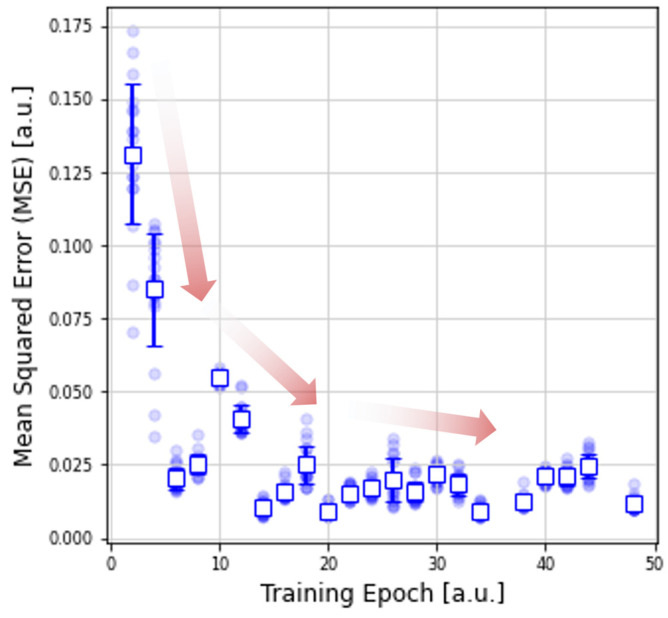
Evaluation metrics of the proposed parameter extraction technique using ANNs. MSE as a function of the training epoch. The MSE is computed between the measured *I-V* loop and the one obtained by simulation using the ANN fitted parameters.

Nonetheless, it is worth pointing out that the previous metric does not necessarily demonstrate that the ANN fitted parameters properly capture the details of the experimental data, and even less quantifies it. For this reason, we evaluated the MSE between the experimental *I-V* data and a simulated *I-V* loop recreated from the calculated fitting parameters. Figure 7 presents these results as a function of the training epoch, showing a steep decrease in the MSE for the first 10 epochs. From the 12th epoch on, the MSE error remains almost constant. In addition, not only does the mean MSE decrease, but so does its dispersion. It is worth pointing out that despite this behavior, the Wasserstein distance shows a rather constant decrease for the entire range of training epochs. This suggests that the MSE might be less sensitive to certain parameters (for instance η_SET_ and η_RESET_). 

**Figure 8:**
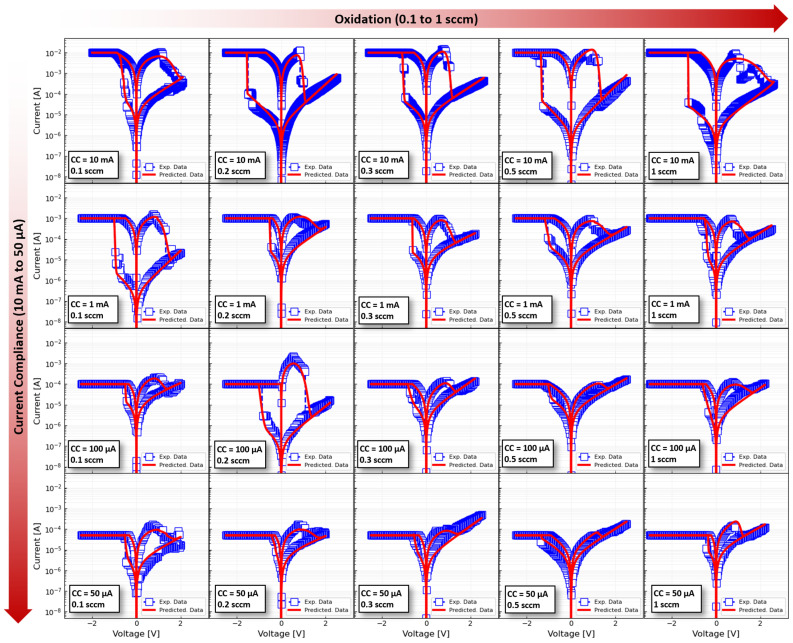
Representative *I-V* loops for each oxidation-current compliance pair (totaling 20 different combinations). Each column from left to right groups the samples fabricated with the same oxidation condition (0.1, 0.2, 0.3, 0.5, 1 sccm) and each row summarizes the measurements carried out with the same current compliance (50 µA. 100 µA, 1 mA, and 10 mA). In each panel, one experimentally measured *I-V* loop is plotted together with a simulation of the DMM whose parameters were obtained with the trained ANN.

A final verification of the suitability of the proposed neural network approach to estimate the DMM parameters is illustrated in Figure 8. In this Figure, the *I-V* loops resulting from running the DMM model with the fitted parameters are superimposed to the experimental input data. Note that the Figure covers the 5 different oxidation cases (from left to right) and the 4 different current compliances (from top to bottom) as well. In this way, both the abrupt transition (digital) observed in the most oxidized samples measured with a high current compliance, as well as the soft transition (quasi-analogue) observed in the least oxidized samples, are captured.

**Figure 9:**
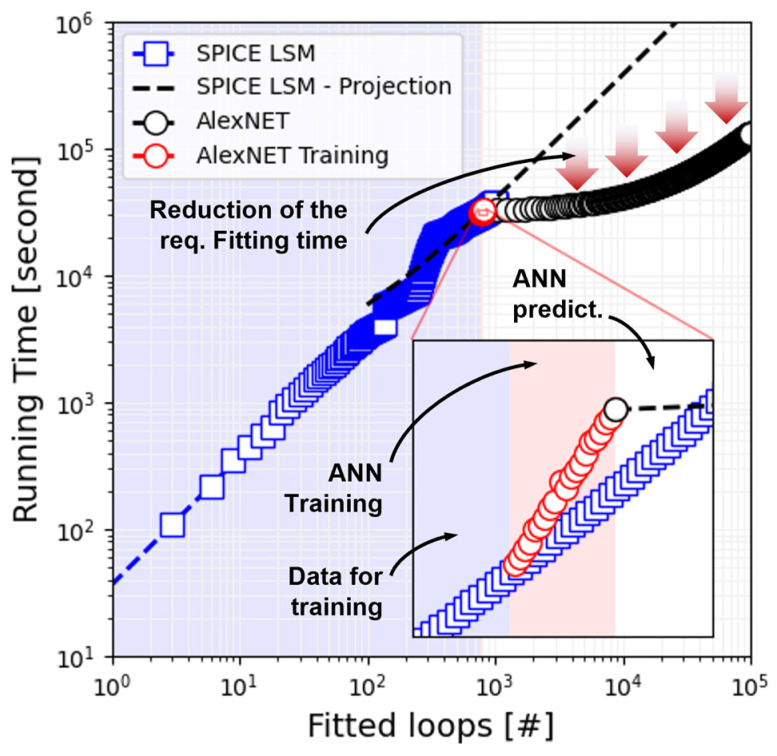
Evaluation metrics of the proposed parameter extraction technique using ANNs. Time required to fit an increasing number of *I-V* loops using the simulator-in-the-loop, iterative approach, and the ANN-based counterpart. For a large number of loops (e.g., 100,000), the ANN approach allows a significant time saving.

Having verified that the DMM fitting parameters are correctly predicted by this ANN-based procedure, we proceeded to quantify the achieved time saving. To do so, we estimated the required time to fit a total of 100,000 *I-V* loops. For the case of the fitting time required for the simulator-in-the-loop approach, we estimated it by considering a linear regression on the fitting-time vs. fitted cycles plot (squared blue markers in Figure 9). We do so because fitting such an amount of data, considering the required time to fit each loop with this method, would take more than 10^6^ s (or up to approximately 11 days). On the contrary, for the case of the fitting time required by the ANN-based approach, we extended our dataset by replicating the dataset roughly 100 times. This is similar to the conventional data augmentation techniques used to reduce the overfitting problems in neural networks, with the difference that the added data is not new. This is not a problem in our case since, at this point, we are not using such dataset for training or testing accuracy, but simply to quantify the time required to forward propagate the information through the network. As shown in Figure 9, the proposed method could potentially reduce the fitting time by more than 1 order of magnitude, even if we consider the required time to generate the training data and the training of the ANN.

It is also worth emphasizing that further time-saving could be achieved by speeding up the training step. Training speed is determined by the learning method and the degree of parallelization (i.e., the hardware used to run the training phase). The learning method employed in this work is the well-known Adam Optimizer, which has become the default learning method regardless of the field and has set the reference in terms of convergence speed (although the Stochastic Gradient Descend method might provide a better generalizing capability). As such, the remaining alternative to speed-up the training is increasing the degree of the parallelization of the hardware used for the training, i.e., using a cluster with a higher number of GPUs [42]. Another option (also exploiting the benefits of parallelization) is the use of FPGA accelerators [43,44,45,46], which can deliver a reduction in the processing time per image of up to 2 orders of magnitude, as reported in Ref. [43]. In that work, an end-to-end FPGA-based CNN accelerator, with all the layers working concurrently in a pipelined structure, remarkably improves the performance.

## 4. Conclusions

In this work, we investigated the use of the AlexNET architecture, a particular case of a convolutional neural network, for extracting the fitting parameters used to model the *I-V* loops from RRAM devices with the Dynamic Memdiode Model (DMM). Experimental data corresponding to devices with different oxygen content and measured with different current compliance levels were considered. In view of this scenario as a case study, we evaluated the impact of the training epochs on the fitting accuracy and quantified it in terms of the relative mean squared error. We proposed a procedure to train the neural network with labeled data—this being *I-V* loops with their associated DMM fitting parameters. This approach was proven to achieve a reduced running time and an increased energy efficiency when fitting many *I-V* loops, which was more than what was typically obtained using the Least-Square-Error minimization method, which needs to be performed individually on each individual measurement.
